# YAP accelerates vascular senescence via blocking autophagic flux and activating mTOR

**DOI:** 10.1111/jcmm.15902

**Published:** 2020-12-12

**Authors:** Xianmei Pan, Bo Wu, Xianglin Fan, Guanghui Xu, Caiwen Ou, Minsheng Chen

**Affiliations:** ^1^ Key Laboratory of Construction and Detection of Guangdong Province Zhujiang Hospital Southern Medical University Guangzhou China; ^2^ Guangdong Provincial Biomedical Engineering Technology Research Center for Cardiovascular Disease Guangzhou China; ^3^ School of Pharmaceutical Science Southern Medical University Guangzhou China

**Keywords:** autophagy, mTOR, vascular senescence, YAP

## Abstract

Yes‐associated protein (YAP), a major effector of the Hippo signalling pathway, is widely implicated in vascular pathophysiology processes. Here, we identify a new role of YAP in the regulation of vascular senescence. The inhibition or deficiency and overexpression of YAP were performed in human umbilical vein endothelial cells (HUVECs) and isolated vascular tissues. Cellular and vascular senescence was assessed by analysis of the senescence‐associated β‐galactosidase (SA‐β‐gal) and expression of senescence markers P16, P21, P53, TERT and TRF1. We found that YAP was highly expressed in old vascular tissues, inhibition and knockdown of YAP decreased senescence, while overexpression of YAP increased the senescence in both HUVECs and vascular tissues. In addition, autophagic flux blockage and mTOR pathway activation were observed during YAP‐induced HUVECs and vascular senescence, which could be relieved by the inhibition and knockdown of YAP. Moreover, YAP‐promoted cellular and vascular senescence could be relieved by mTOR inhibition. Collectively, our findings indicate that YAP may serve as a potential therapeutic target for ageing‐associated cardiovascular disease.

## 1 INTRODUCTION

Senescence, one of the main risk factors contributing to vascular dysfunction and the progression of vascular diseases,^1^, ^2^ is characterized by the gradual decline in physiological functions occurring at both cellular and organic levels.^3^ Cellular senescence is known as replicative senescence, with an irreversible cell cycle arrest characteristic, while organic senescence is a kind of reduced stress response and increases homoeostatic imbalance, resulting in a variety of disorders, including cardiovascular disease, obesity, diabetes, neurodegeneration and neoplastic diseases.^3^, ^4^ DNA damage caused by intrinsic and extrinsic stress factors can induce premature senescence and activate many markers similar to those in replicative senescence, known as “stress‐induced premature senescence”.^5^

Yes‐associated protein (YAP), a major effector of the Hippo signalling pathway, plays important roles in the regulation of development, homoeostasis, regeneration and so forth. YAP can translocate into the nucleus and interact with transcription factors to regulate the expression of target genes when the Hippo pathway is inactive. Whereas when the Hippo pathway is active,YAP is phosphorylated, resulting in cytoplasmic retention and proteolytic degradation.^6^, ^7^, ^8^ Previous studies have discovered that YAP exerts important effects in the regulation of cellular senescence, in addition to cell proliferation and apoptosis, but the effects vary with different cells.^9^, ^10^, ^11^, ^12^ Additionally, YAP is found widely implicated in vascular physiopathology processes,^13^, ^23^ such as modulating the activation of endothelial cellular and vascular inflammation, the hallmark of senescence.^14^, ^15^

Recently, it has been found that YAP could activate the mammalian target of rapamycin (mTOR), a critical regulator of regulating life span and ageing.^3^, ^16^ In addition, many studies indicated that YAP modulates autophagy by interacting with TANK‐binding kinase 1(TBK1) in the cytoplasm or regulating Armus and other members of this family, which plays a major role in senescence process.^17^, ^18^, ^19^, ^20^, ^21^, ^22^, ^23^, ^24^ However, whether autophagy and mTOR signalling pathway are involved in the senescence promoting effect of YAP remains unclear. Here, we identify a novel function of YAP in regulation of vascular senescence and investigate the potential mechanism underlying the senescence promoting effect.

## 2 MATERIALS AND METHODS

### 2.1 Cell culture

HUVECs were cultured in 4.5 g/L Dulbecco's modified Eagle's medium (DMEM) with 10% foetal bovine serum and 1% antibiotics in a 5% CO_2_ atmosphere at 37°. Lipopolysaccharide (LPS, Abcam, USA) was used to induce cellular senescence. After incubating (6 × 10^6^ cells per well, 6‐well plate) with DMEM for 12 hours, HUVECs were cultured for 6 days in 0.5 μg/mL LPS (DMEM with 5% foetal bovine serum and 1% antibiotics in a 5% CO_2_ atmosphere at 37°C).

### 2.2 Silencing RNA knockdown and adenovirus infection

The YAP adenovirus (Ad‐YAP, pAdeno‐MCMV‐HA‐YAP‐IRES2‐EGFP) and negative control adenovirus (pAdeno‐MCMV‐3Flag‐IRES2‐EGFP) were designed and constructed by Obio technology (Shanghai, China), and the transfection was performed following manufacturer's protocol. YAP siRNA (siYAP; Obio technology; China) of 50 pmol and Lipofectamine^®^ 3000 reagent (Life Technologies) of 2 μL were, respectively, added to 150 μL medium without serum and antibiotic and incubated at room temperature for 5 minutes. The above 2 solutions were mixed well for 20 minutes at room temperature. Then, cells were incubated with the transfection complex solution for 6 hours at 37°C, and re‐incubated in complete medium for an additional 18 hours at 37°C. The synthetic sequence of siYAP is as follows: siYAP#1:GCGTAGCCAGTTACCAACA; siYAP#2:CAGTGGCACCTATCACTCT; siYAP#3:GGTGATACTATCAACCAAA. Control siRNA is a non‐targeting 20‐25 nt siRNA (Scramble; Obio technology; China) designed as a negative control.

### 2.3 Animal experiments

Animal experiments were approved by the Institutional Animal Care and Use Committee at Southern Medical University and carried out in accordance with the UK Animals Act, 1986 and associated guidelines. All animals used in this experiment were specific pathogen‐free Sprague‐Dawley (SD) rats from the Laboratory Animal Research Center of Southern Medical University (Guangzhou, China) and were maintained in an air‐conditioned room under controlled lighting, temperature and humidity with free access to standard rodent chow and water.

Animas treated with Ad‐YAP: 4‐week‐old SD rats first were randomly assigned to the following four groups (n = 4 for each group): 0 PFU group, 6.32 × 10^4^ PFU group, 6.32 × 10^5^ PFU group and 12.64 × 10^5^ PFU group. Next, the rats in control group were treated with phosphate‐buffered saline (PBS), while the rats in the Ad‐YAP group were treated with PBS and Ad‐YAP in the tail vein. Finally, rats were killed under anaesthesia by pentobarbital sodium and the section of aorta from the ascending aorta to the common iliac arteries was dissected after 4 weeks.

Culture and stimulation of vascular tissues: the vascular tissues were isolated from rats weighting 220‐250 g, and then, the vascular tissues were cultured for 6 days in 0.5 μg/mL LPS (DMEM with 5% foetal bovine serum and 1% antibiotics in a 5% CO2 atmosphere at 37°) to induce vascular senescence. Transfection with Ad‐YAP: YAP adenovirus (Ad‐YAP, pAdeno‐MCMV‐HA‐YAP‐IRES2‐EGFP) was performed following manufacturer's protocol.

### 2.4 Immunofluorescence staining

HUVECs were seeded into coverslips and first permeabilized with 0.1% Triton X‐100 after fixed with 4% paraformaldehyde for 30 minutes. Then, the cells were washed and blocked with 1% BSA for 30 minutes. Next, the cells were incubated overnight at 4°C with a primary antibody: anti‐YAP (1:100, Boster, China) and anti‐P53 (1:100, Abcam, USA). After incubation with secondary antibody (1:100, Bioworld Technology, China) at room temperature for 2 hours, the cell nuclei were stained with DAPI (Beyotime, China, 1 μg/mL). Immunofluorescence was detected by using confocal laser scanning microscope (Leica sp8, Germany).

### 2.5 Western Blot analysis

Total protein was extracted from HUVECs or isolated vascular tissue using RIPA buffer with protease and phosphatase inhibitors. Nuclear and cytoplasmic protein were extracted from HUVECs using Nuclear Extraction Kit (BestBio BB‐3102, China). Protein concentrations were measured via BCA™ Protein Assay Kit (Thermo Fisher Scientific, USA). Protein was separated by sodium dodecyl sulphate‐polyacrylamide gel electrophoresis (SDS‐PAGE) and transferred to PVDF membranes. PVDF membranes were blocked by 5% skim milk in Tris‐buffered saline at room temperature for 2 hours and then incubated with the following primary antibodies overnight at 4°C: anti‐p16, anti‐P21, anti‐P53 (Abcam, USA); anti‐YAP, anti‐PYAP (Boster, china); anti‐mTOR, anti‐P‐mTOR (Ser2448), anti‐P62, anti‐Beclin1, anti‐LC3B (Cell Signaling Technology, USA); anti‐β‐actin (Bioworld technology, China); and anti‐β‐tubulin, anti‐LaminB (wanleibio, China). All primary antibodies were diluted in 1:1000. The membranes next were washed and incubated with secondary antibodies (1:8000, Bioworld technology, China) at room temperature for 2 hours. Protein signals on the bands were visualized by the enhanced chemiluminescence (Thermo Fisher Scientific, Waltham, USA). Protein expression levels were quantified by densitometry using ImageJ 64 software, and the relative protein expression was compared with β‐actin.

### 2.6 SA‐β‐gal staining

The senescence Cells Histochemical Staining Kit (Beyotime C0602) was used to evaluate senescence following manufacturer's instructions. Firstly, cells or tissues were incubated with fixation buffer for 10 minutes at room temperature. And then, samples were washed and incubated with staining mixture (containing X‐gal) at 37°C for 24‐30 hours. Senescence degree was next detected by a fluorescence microscope (Leica Dmi8, Germany). Finally, the numbers of SA‐β‐gal‐positive cells were counted by ImageJ software in a blinded manner.

### 2.7 Evaluation of fluorescent LC3 puncta

Adenovirus of tandem fluorescent mRFP‐GFP‐LC3 was designed and constructed by Hanbio Biotechnology (Shanghai, China). After transfection with mRFP‐GFP‐LC3, autophagosomes (AP) were labelled yellow (mRFP and GFP) whereas autolysosomes (AL) were labelled red (mRFP only). Briefly, HUVECs firstly were cultured on coverslips and then treated with adenovirus of tandem fluorescent mRFP‐GFP‐LC3(MOI = 15) for 24 hours, and cells were then washed with PBS, fixed with 4% paraformaldehyde, mounted with a reagent containing DAPI as the method in previous study^25^ and detected using confocal laser scanning microscope (Leica sp8, Germany). Lastly, the numbers of APs (yellow dots) and ALs (red dot) were counted manually.

### 2.8 Statistical analysis

All data were analysed by SPSS 21.0 software and expressed as mean ± standard error of the mean (SEM). Statistical differences were evaluated using Student's test or one‐way analysis of variance (ANOVA), followed by the Tukey‐Kramer HSD post hoc test for multiple comparisons. *P* < 0.05 was considered as significant statistically differences.

## 3 RESULTS

### 3.1 YAP is highly expressed in old vascular tissues

**Figure 1:**
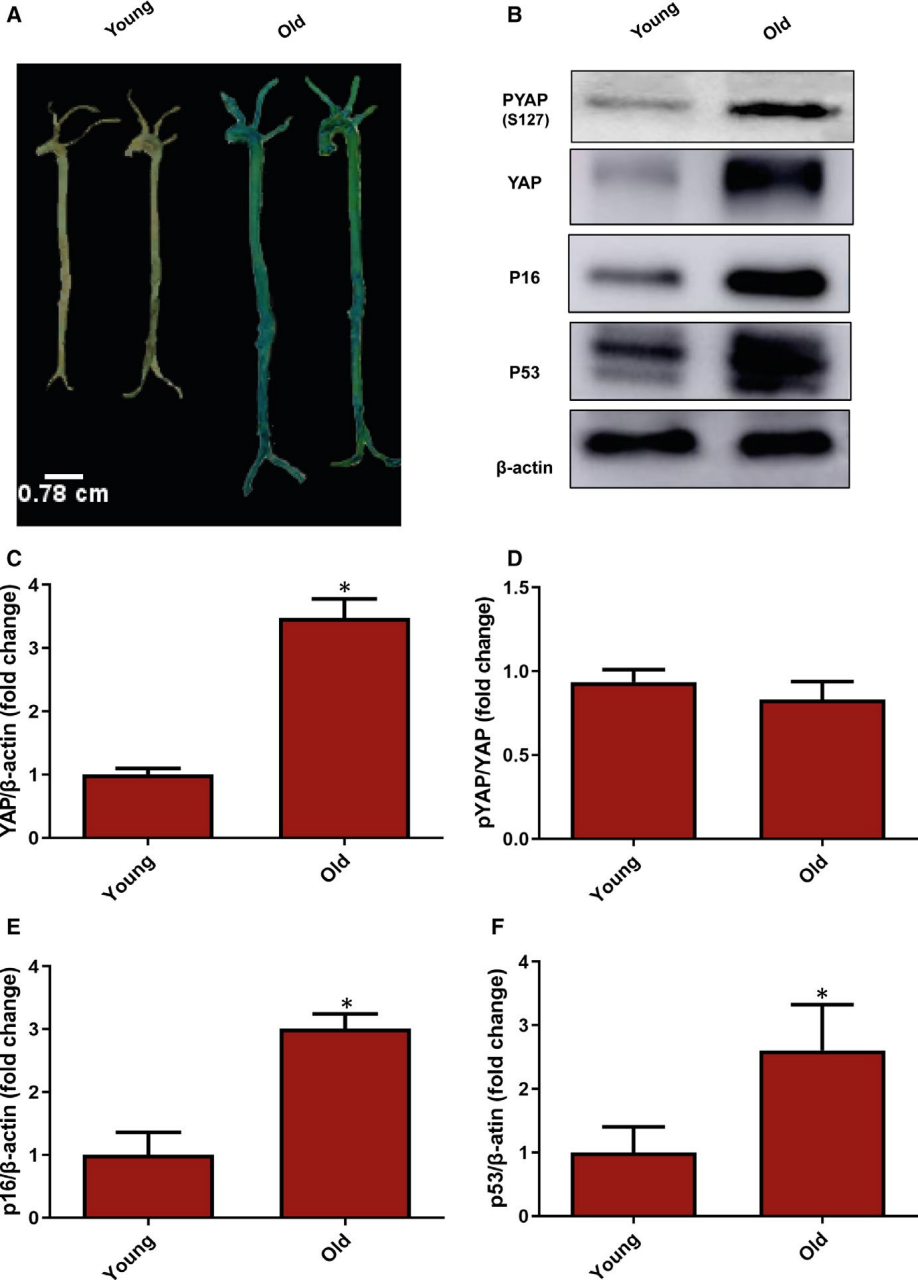
Expression of YAP in young and old vascular tissues. A, SA‐β‐gal staining was performed to analyse vascular senescence degree from ageing (13 mo) and young (1 mo) rats. B, Western blot was used to analyse the expression of PYAP and YAP and senescence markers P16 and P53 in vascular tissues from ageing and young rats. C, The semi‐quantification of YAP. D, The semi‐quantification of PYAP. E, The semi‐quantification of P16. F, The semi‐quantification of P53. All experiments were repeated three times, and data are expressed as mean ± SEM. *P < 0.05 vs young group

To determine the difference in YAP expression, the vascular tissues from young and ageing rats were attained. SA‐β‐gal staining was firstly used to examine the senescence in vascular tissues. As shown in Figure 1A, vascular tissues obtained from ageing rats showed significantly higher β‐gal staining than those from young rats. Then, senescence markers P16 and P53 were used to determined vascular senescence by Western blot and the results showed that P16 and P53 were elevated significantly in old vascular tissue (Figure 1B,E‐F). Interestingly, we found YAP was highly expressed in old vascular tissues (Figure 1B‐D). Taken together, our results suggest that YAP expression has positive correlation with vascular senescence.

### 3.2 Inhibition of YAP pharmacologically or genetically attenuates cellular and vascular senescence

**Figure 2:**
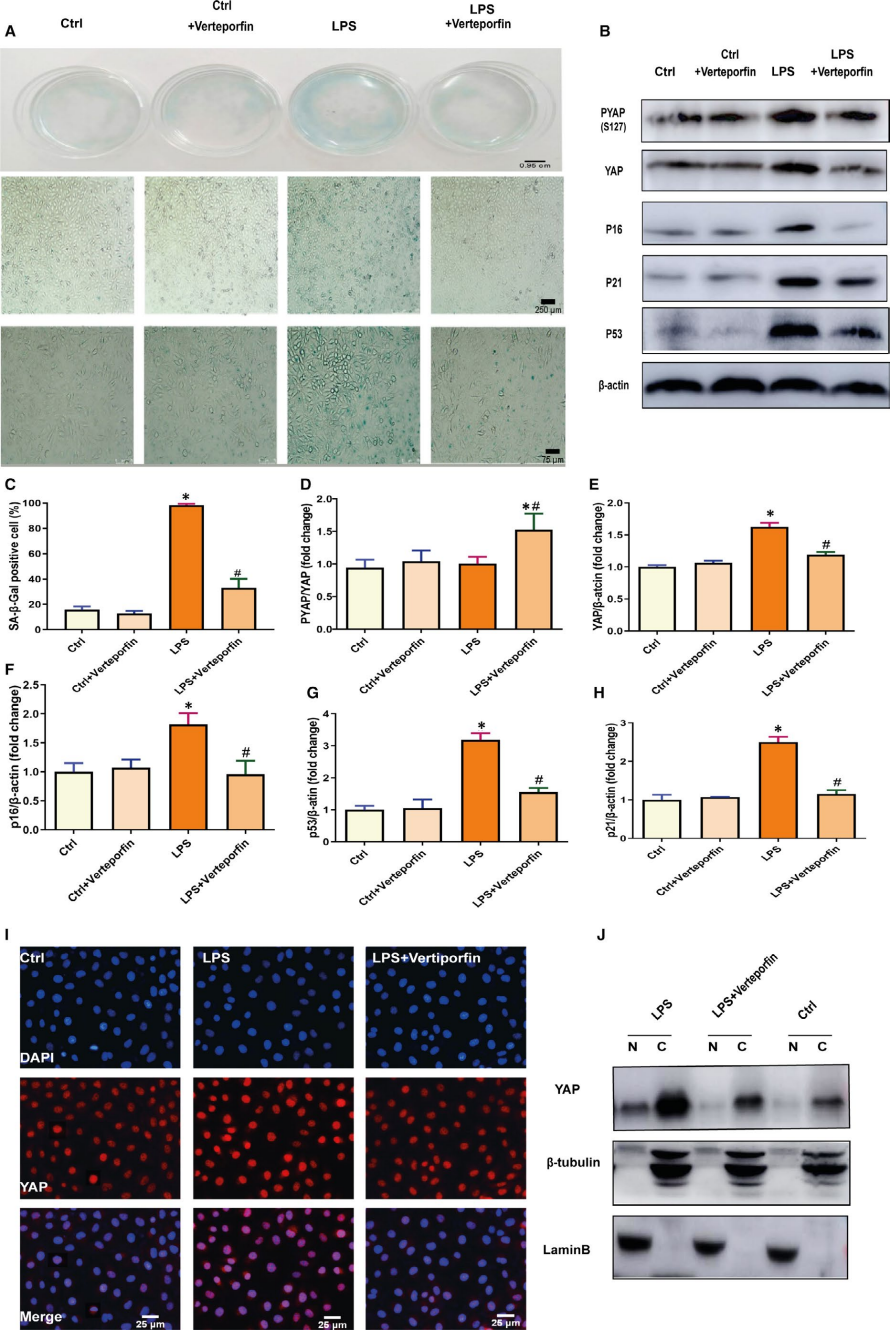
Inhibition of YAP pharmacologically attenuates cellular senescence. A, To build a cellular senescence, HUVECs were cultured for 6 days in 0.5 μg/mL LPS after incubating with DMEM for 12 h. Cellular senescence was assessed by analysis of SA‐β‐gal in HUVECs treated with 0.1 mg/mL verteporfin for 6 d. B, Western blot was preformed to analyse PYAP, YAP, P16, P21 and P53 in HUVECs treated with or without 0.1 mg/mL verteporfin in the presence of 0.5 μg/mL LPS for 6 d. C, SA‐β‐gal‐positive cell percentage. D‐H, The semi‐quantification of the proteins in panel C, respectively. I, Immunofluorescence attaining was used to ascertain the localization of YAP. The nuclei were labelled with DAPI (blue staining); YAP is red (IgG(H + L)Cy3). J, Expression of indicated proteins was analysed by immunoblot in nuclear and cytoplasmic protein extractions from HUVECs treated with or without 0.1 mg/mL verteporfin in the presence of 0.5 μg/mL LPS for 6 d. All experiments were repeated at least three times, and data are expressed as mean ± SEM, *P < 0.05 vs Ctrl group, ^#^ *P* < 0.05 vs LPS group

To evaluate the potential roles of YAP in vascular senescence, we first treated HUVECs with YAP inhibitor verteporfin, a compound reported to interfere with YAP binding to the TEAD transcription factors which interact with coactivator YAP and mediate downstream gene expression.^11^, ^26^ To build a model of cellular and organic senescence, the stressor used in this study was LPS, an experimental stressor has been found to induce oxidative stress senescence and accelerate the expression of YAP in human and mouse endothelial cells.^27^ Our results showed that HUVECs treated with LPS exhibited significantly increased β‐gal‐positive cells than the control group, and cells treated with LPS and verteporfin showed significantly decreased β‐gal‐positive cells than LPS group (Figure 2A,C). In addition, HUVECs treated with LPS showed significantly up‐regulation of YAP and senescence markers P53, P21 and P16, while cells treated with LPS and verteporfin displayed significantly down‐regulation of YAP, P53, P21 and P16 (Figure 2B,D‐H). Since nuclear YAP plays a critical role in regulating its target genes to exert biological functions, immunofluorescence staining showed that LPS augmented the localization of YAP into the nucleus, which could be inhibited by verteporfin (Figure 2I‐J). Collectively, these results illustrated that inhibiting YAP pharmacological activity decreases cellular senescence.

**Figure 3:**
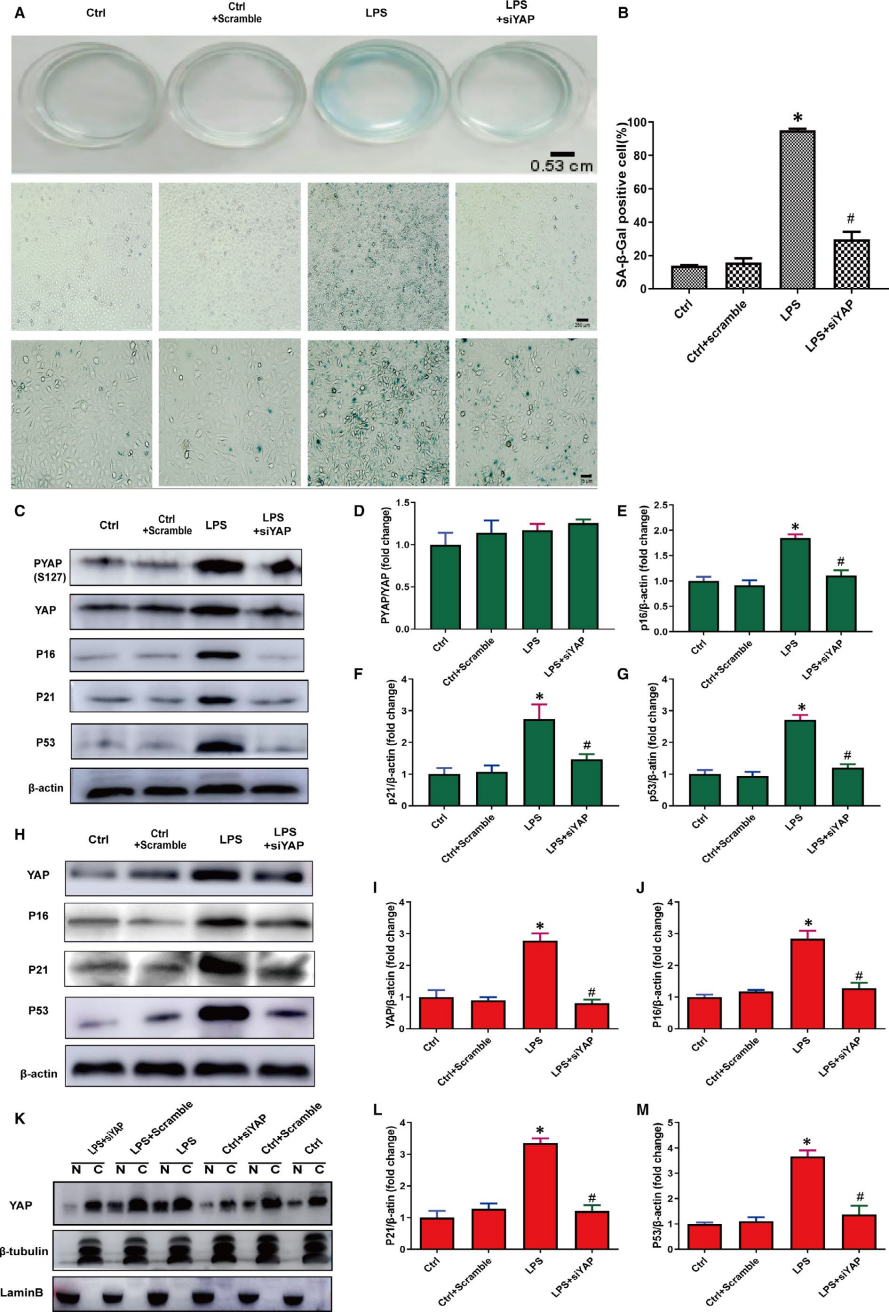
Knockdown of YAP inhibits cellular and vascular senescence. A, SA‐β‐gal staining was used to analyse senescence degree in HUVECs transfected with YAP siRNA (siYAP#1, siYAP#2, siYAP#3) or negative control siRNA (non‐targeting 20‐25 nt siRNA) for 6 h. B, SA‐β‐gal‐positive cell percentage. C, Western blot was preformed to analyse PYAP, YAP, P16, P21 and P53 in cells transfected with YAP siRNA (siYAP#1, siYAP#2, siYAP#3) or negative control siRNA for 6 h. D‐G, The semi‐quantification of the proteins in panel C respectively. H, Western blot was used to analyse YAP and senescence markers P16, P21 and P53 in vascular tissues treated with YAP siRNA (siYAP#1, siYAP#2, siYAP#3) or negative control siRNA for 6 h. I‐J, The semi‐quantification of the proteins in panel H, respectively. K, Expression of indicated proteins was analysed by immunoblot in nuclear and cytoplasmic protein extractions from in HUVECs treated with or without YAP siRNA (siYAP#1, siYAP#2, siYAP#3) or negative control siRNA for 6 h. L‐M, The semi‐quantification of the proteins in panel H, respectively. All experiments were repeated at least three times, and data are expressed as mean ± SEM, *P < 0.05 vs Ctrl group, ^#^ *P* < 0.05 vs LPS group

We next delivered siRNA specifically targeting YAP in HUVECs to further assess the effects of YAP on senescence. We found HUVECs treated with YAP siRNA showed decreased β‐gal staining (Figure 3A‐B) and significantly down‐regulation of YAP, P53, P21 and P16 (Figure 3C‐G,K). In addition, LPS + scramble group showed increased senescence markers similarly to LPS, and the Ctrl and Ctrl + scramble showed similar senescence (Figure S1A‐C). These results suggesting YAP knockdown attenuated the senescence of HUVECs. We also performed similar experiments in vascular tissues and found that the vascular tissues treated with YAP siRNA expressed lower expression of YAP, P53, P21 and P16 (Figure 3H‐J,L‐M), indicating that YAP knockdown inhibited vascular senescence.

### 3.3 Overexpression of YAP accelerates cellular and vascular senescence

**Figure 4:**
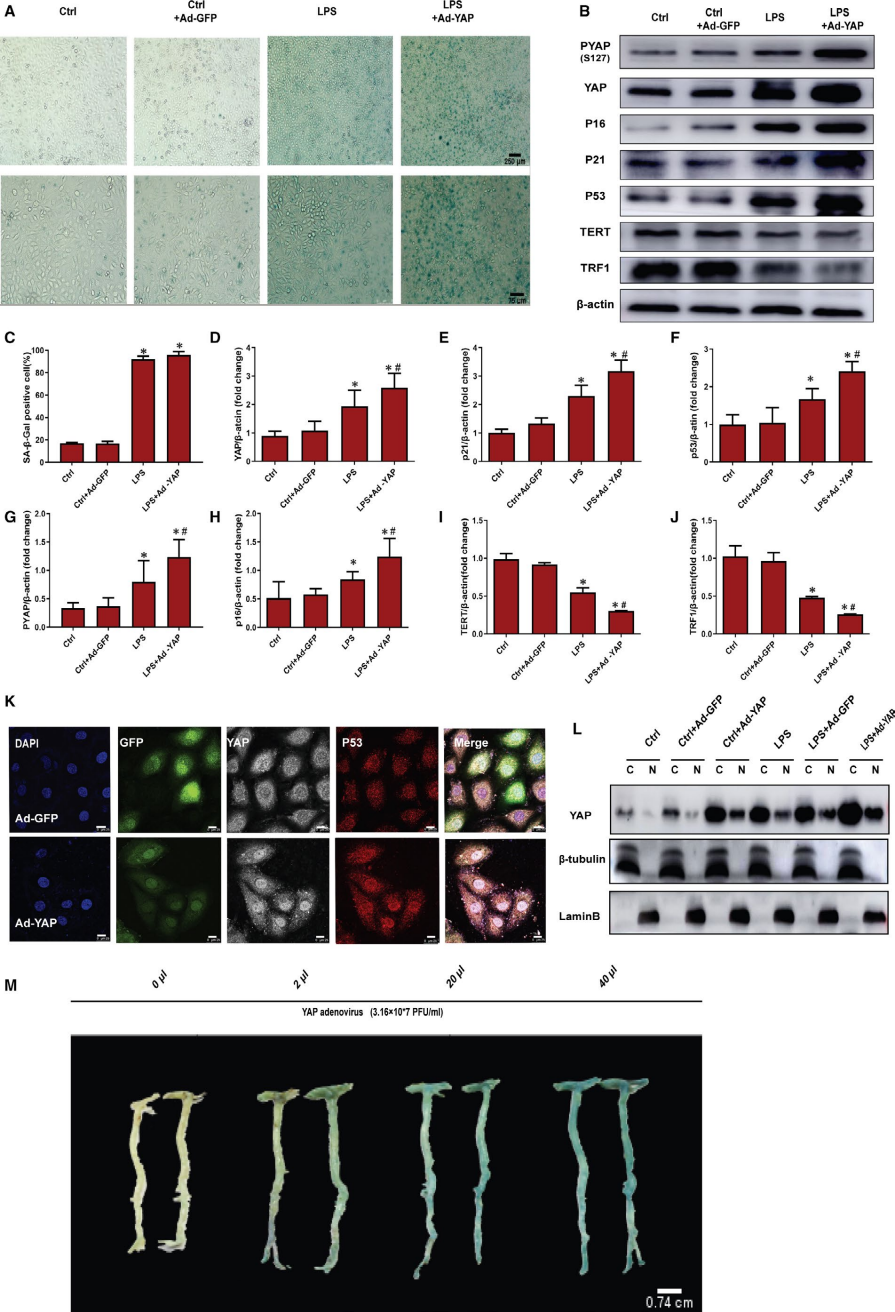
YAP accelerates cellular and vascular senescence. A, SA‐β‐gal staining was used to analyse senescence degree in HUVECs treated with 0.5 μg/mL LPS for 6 d or Ad‐YAP for 12 h or co‐treated with both. B, Western blot was used to analyse PYAP, YAP, P16, P21, P53, TERT and TRF1 in HUVECs treated 0.5 μg/mL LPS for 6 d or Ad‐YAP for 12 h or co‐treated with both. C, SA‐β‐gal‐positive cell percentage in plane A. D‐J, The semi‐quantifications of the proteins of plane B, respectively. K, Immunofluorescence attaining was used to ascertain the localization of YAP and P53 in HUVECs treated with Ad‐GFP or Ad‐YAP for 12 h. The nuclei were labelled with DAPI (blue staining; P53 are red (IgG(H + L)Cy3); YAP is white (Alexa Fluor 647). Scale bar = 25 μm. L, Expression of indicated proteins was analysed by immunoblot in nuclear and cytoplasmic protein extractions from HUVECs treated with 0.5 μg/mL LPS for 6 d or Ad‐YAP for 12 h or co‐treated with both. M, Rats were treated with YAP adenovirus in different concentration gradients for 4 wk. SA‐β‐gal staining was used to analyse vascular senescence degree. All experiments were repeated at least three times, and data are mean ± SEM,*P < 0.05 vs Ctrl group, ^#^ *P* < 0.05 vs LPS group

To ascertain the effect of YAP overexpression on senescence, Ad‐YAP was delivered into HUVECs. We found HUVECs co‐treated with Ad‐YAP showed more β‐gal staining (Figure 4A,C) and displayed significantly higher expression of YAP and senescence markers P53, P21 and P16 (Figure 4B,D‐H,K‐L). Additionally, Western blot experiment was performed to determine the telomere activity, a well‐accepted marker of cellular senescence, which was affected by YAP. The telomere structure and length stabilization were associated with telomerase reverse transcriptase (TERT) and telomeric‐associated protein TRF1.^28^ Our results showed HUVECs treated with Ad‐YAP displayed significantly lower TERT and TRF1 than LPS group and control group (Figure 4B,I‐J). Furthermore, we added different concentrations of Ad‐YAP into rats to determine the effects of YAP overexpression on vascular senescence. As shown in Figure 4E, β‐gal staining fold change was related to Ad‐YAP concentrations and the vascular tissue size changed in a concentration‐dependent manner (Figure 4M), which was consistent with prior studies that YAP plays an important role in cell proliferation and organism size.^11^ Collectively, those in vitro and in vivo results supported that overexpression of YAP accelerates cellular and vascular senescence.

### 3.4 Autophagy and mTOR are involved in YAP promoting cellular and vascular senescence

**Figure 5:**
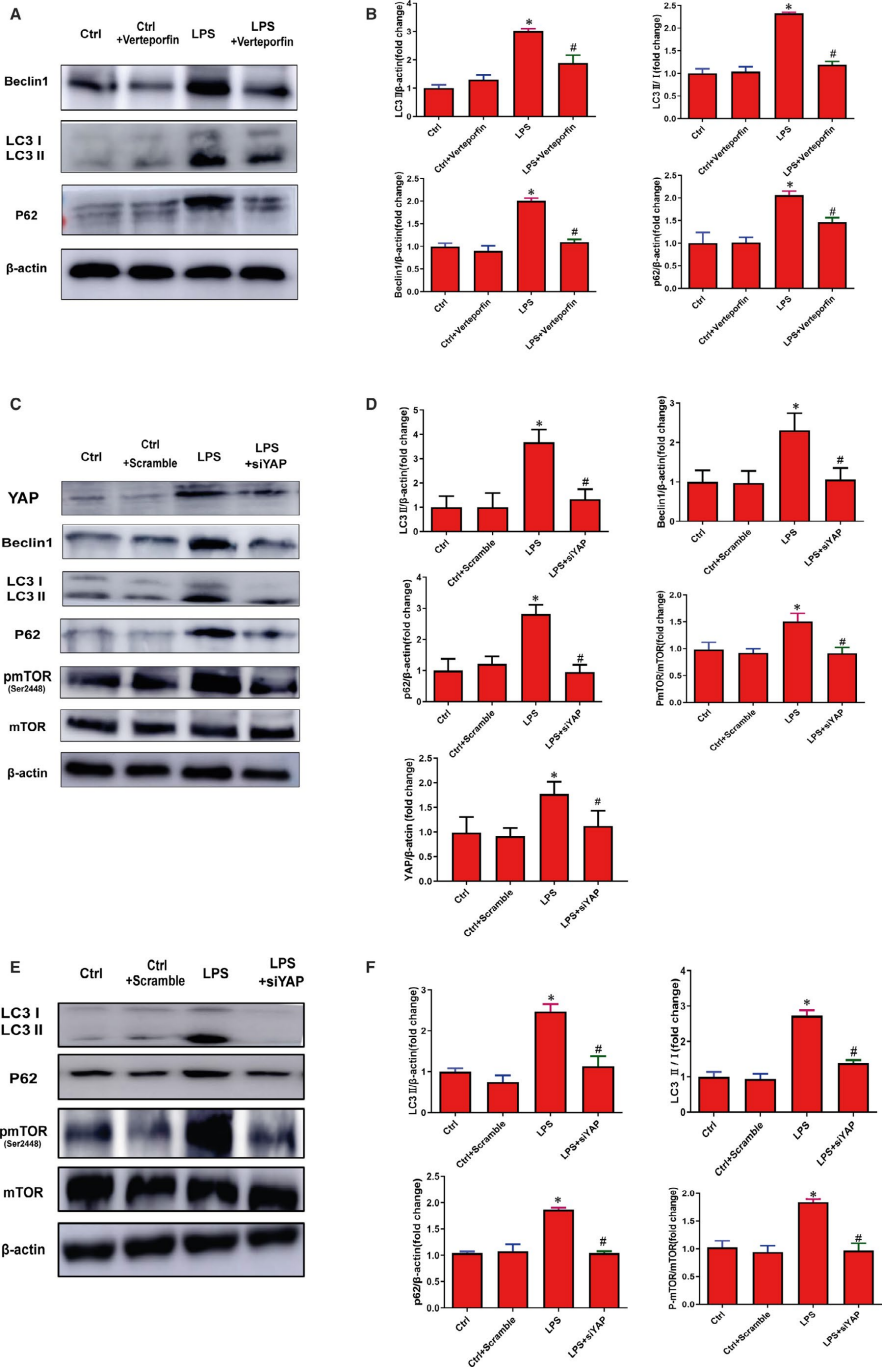
Impaired autophagy occurred during YAP promoting cellular and vascular senescence. A, Western blot was preformed to analyse the expression of Beclin1, LC3Ⅱ, LC3Ⅰ and P62 in HUVECs treated with or without 0.1 mg/mL verteporfin in the presence of 0.5 μg/mL LPS for 6 d. B, The semi‐quantifications of the proteins of plane A, respectively. C, Western blot was used to analyse YAP, p‐mTOR (Ser2448), mTOR, Beclin1, LC3Ⅱ, LC3Ⅰ and P62 expression in HUVECs with or without YAP siRNA (siYAP#1, siYAP#2, siYAP#3 for 6 h) in the presence of LPS (0.5 μg/mL LPS for 6 d). D, The semi‐quantifications of the proteins of plane C, respectively. E, Western blot was preformed to analyse p‐mTOR (Ser2448), mTOR, LC3Ⅱ, LC3Ⅰ and P62 in isolated vascular tissue with or without YAP siRNA (siYAP#1, siYAP#2, siYAP#3 for 6 h) in the presence of LPS (0.5 μg/mL for 6 d). F, The semi‐quantifications of the proteins of plane E, respectively. All experiments were repeated at least three times, and data are expressed as mean ± SEM, *P < 0.05 vs Ctrl group, ^#^ *P* < 0.05 vs LPS group

**Figure 6:**
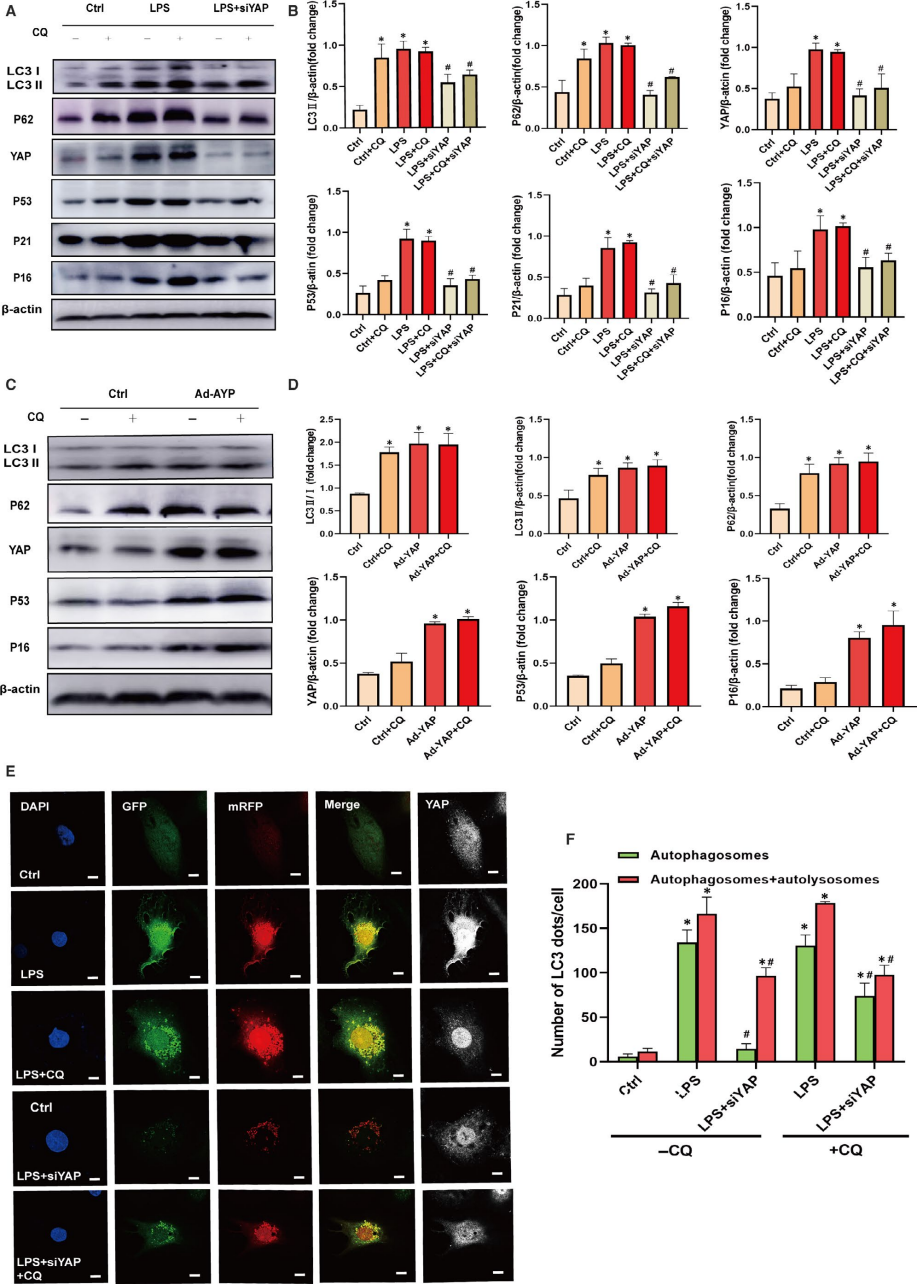
Autophagic flux was impaired during YAP promoting cellular senescence. A, Western blot was preformed to analyse the expression of LC3Ⅱ, LC3Ⅰ, P62, YAP, P53, P21 and P16 in HUVECs co‐treated with or without Chloroquine (CQ, 10 mol/L for 24 h) in the presence of YAP siRNA (siYAP#1, siYAP#2, siYAP#3 for 6 h). Experiments were repeated three times. B, The semi‐quantifications of the proteins of plane A, respectively. Data are expressed as mean ± SEM, *P < 0.05 vs Ctrl group, ^#^ *P* < 0.05 vs LPS group. C, Western blot was preformed to analyse the expression of LC3Ⅱ, LC3Ⅰ, P62, YAP, P53 and P16 in HUVECs co‐treated with or without CQ (10 mol/L for 24 h) in the presence of Ad‐YAP. All experiments were repeated at least three times. D, The semi‐quantifications of the proteins of plane C, respectively. Data are expressed as mean ± SEM, *P < 0.05 vs Ctrl group, ^#^ *P* < 0.05 vs Ad‐YAP group. E, Tandem fluorescent mRFP‐GFP‐LC3 adenovirus was subjected to HUVECs to detect the numbers of APs in the presence and absence of CQ (10 mol/L) for 24 h. The nuclei were labelled with DAPI (blue staining); GFP dots are green; mRFP dots are red; YAP is white (Alexa Fluor 647). Scale bar = 10 μm. Experiments were repeated three times. F, Quantitative analysis of APs (yellow dots) and ALs (red dot) in plan E by counting manually; N = 30‐50 nuclei per group. Data are expressed as mean ± SEM,*P < 0.05 vs Ctrl group, ^#^ *P* < 0.05 vs LPS group

Previous studies have discovered that YAP regulates autophagy by actin depolymerization to control proliferation.^22^, ^29^ Therefore, the level of autophagy was examined by Western blot in HUVECs. The results revealed that HUVECs treated with LPS showed up‐regulation of Beclin1, LC3Ⅱ, LC3Ⅱ/Ⅰ and P62, while inhibiting YAP pharmacologically and genetically decreased the expression of Beclin1, LC3Ⅱ, LC3Ⅱ/Ⅰ and P62 (Figure 5A‐D). Similar results were observed in vascular tissue treated with YAP siRNA (Figure 5E‐F). Next, we examined the autophagic flux by detecting the numbers of autophagosomes (APs) and autolysosomes (ALs) in the presence and absence of chloroquine, which inhibits lysosomal acidification and prevents autophagosome‐lysosome fusion.^30^ We found HUVECs treated with Ad‐YAP displayed similar effects to LPS, including accumulated APs and few ALs accompanying with higher expression of YAP, P53, P21 and P16. However, cells co‐treated with chloroquine showed no alterations with significance in the numbers of APs and ALs and the expression of YAP, P53, P21 and P16 (Figure 6A‐F), suggesting that autophagic flux blockage occurs during YAP promoting senescence. In addition, we found that YAP knockdown attenuated autophagic flux blockage in HUVECs, with more ALs, less APs and decreased expression of YAP, P53, P21 and P16 of (Figure 6A‐B,E). Collectively, these findings showed that autophagic flux blockage occurred during YAP promoting senescence, which can be relieved by YAP inhibition.

**Figure 7:**
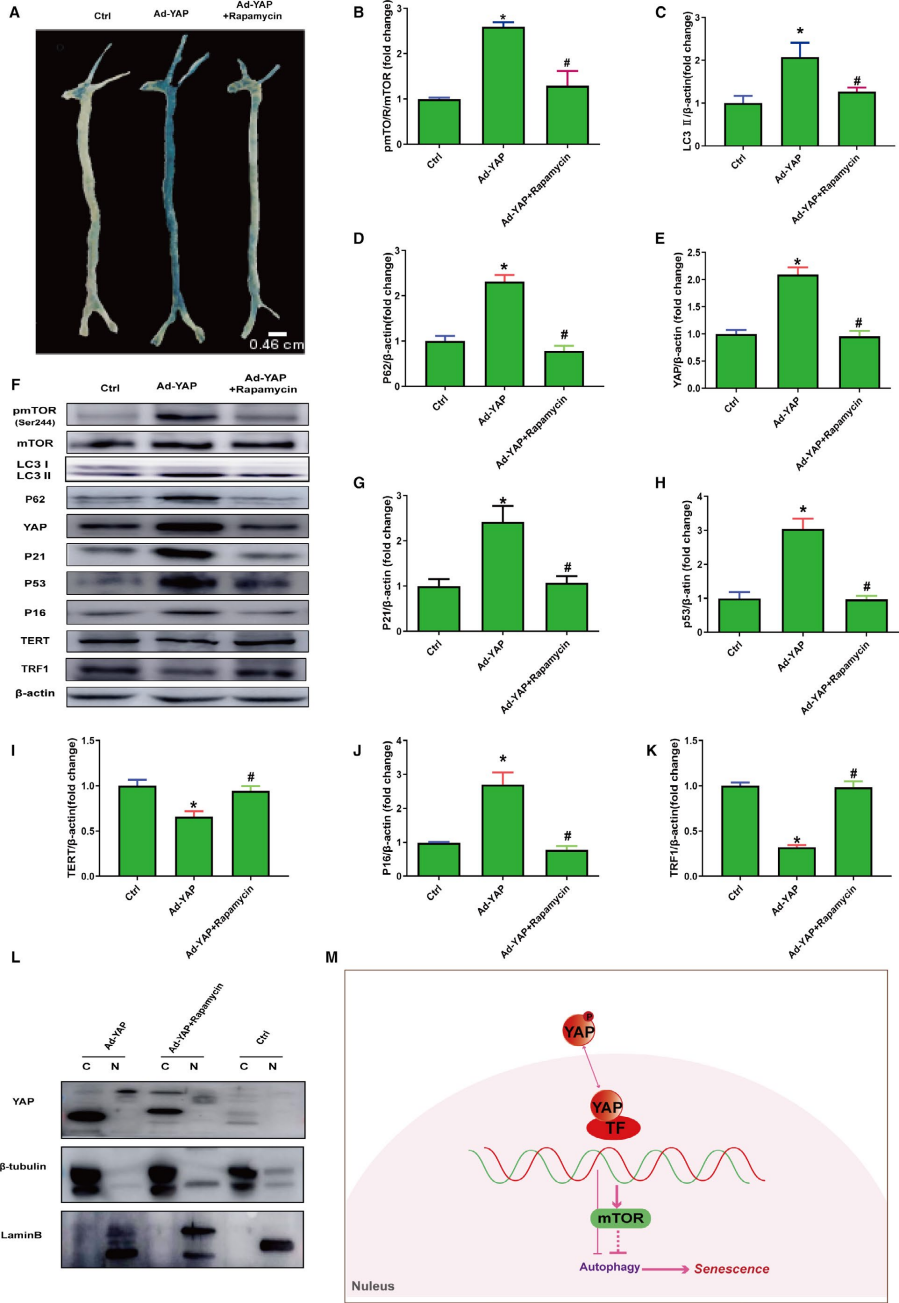
mTOR activity is required in YAP‐promoted cellular and vascular senescence. A, SA‐β‐gal staining was used to analyse senescence degree in vascular tissues co‐treated with or without Rapamycin (5 mol/L for 24 h) in the presence of Ad‐YAP. B‐E, The semi‐quantifications of the proteins in panel F, respectively. F, Western blot was used to analyse p‐mTOR (Ser2448), mTOR, P62, LC3Ⅰ, LC3Ⅱ, YAP, P16, P21, P53, TERT and TRF1 in HUVECs co‐treated with or without rapamycin in the presence of YAP adenovirus. G‐K, The semi‐quantifications of the proteins in panel F, respectively. L, Expression of indicated proteins was analysed by immunoblot in nuclear and cytoplasmic protein extractions from HUVECs co‐treated with or without rapamycin (5 mol/L for 24 h) in the presence of Ad‐YAP. M, Illustration of mechanisms in regulating vascular senescence by YAP. YAP accelerates vascular senescence via mTOR in that autophagy may involve. Arrows indicate facilitation, and horizontal lines indicate suppression. All experiments were repeated three times, and data are expressed as mean ± SEM, *P < 0.05 vs Ctrl group, ^#^ *P* < 0.05 vs Ad‐YAP group

Noticeably, prior works had proved that mTOR pathway plays an important role in regulating life span and ageing.^3^ In addition, YAP could regulate mTOR pathway by PTEN, an upstream negative regulator of mTOR.^16^, ^31^, ^32^, ^33^ Herein, we investigated mTOR signal activity in YAP promoting senescence and found that HUVECs or vascular tissue treated with siRNA‐YAP showed down‐regulated ratio of p‐mTOR to mTOR (Figure 5C‐F), and cells treated with Ad‐YAP showed up‐regulated ratio of p‐mTOR to mTOR (Figure 7B,F). Then, we inhibited mTOR activity with rapamycin to ascertain the roles of mTOR in YAP promoting senescence. The results showed that vascular tissues treated with rapamycin displayed less β‐gal staining with significance (Figure 7A), and lower expression of YAP, P53, P21, P16, TERT and TRF1 (Figure 7E‐L), suggesting that inhibition of mTOR activity down‐regulates YAP expression and partially rescued YAP‐induced senescence. In addition, the autophagic flux blockage was partly relieved by mTOR inhibition (Figure 7C‐D,F). Taken together, these findings illustrated that mTOR activity is essential for YAP promoting senescence.

## 4 DISCUSSION

This study verified the important role of YAP in regulating vascular senescence for the first time. Here, we found that: (a) YAP was highly expressed in old vascular tissues and its level was correlated with the up‐regulation of senescence markers including P16 and P53; (b) inhibition of YAP pharmacologically or genetically decreased senescence, while overexpression of YAP increased the senescence in both HUVECs and vascular tissues; (c) autophagic flux blockage and mTOR signal activation occurred during YAP‐induced senescence, which could be relieved by the inhibition and knockdown of YAP in HUVECs and vascular senescence. (d) YAP‐promoted cellular and vascular senescence could be rescued by mTOR inhibition.

Prior works had documented the effectiveness of YAP in cellular senescence. However, the effects of YAP on senescence are quite different in various cells. For example, some scholars found that down‐regulation of YAP in IMR90 tumour cells increased cell senescence ^11^; others also found that up‐regulation of YAP in Werner syndrome‐derived fibroblasts could accelerate cellular senescence,^10^ which is consistent with our study that up‐regulation of YAP in HUVECs and vascular tissues accelerates senescence. It comes to the key question of our study—how YAP modulates cellular and vascular senescence.

Autophagy is a complex intracellular dynamic process that delivers cytoplasmic constituents for degradation into lysosomes,^34^ which is essential in mediating proper vascular function, as its role in cell survival and smooth muscle cell phenotype and proliferation.^35^ In addition, it was reported that autophagic flux is impaired during senescence‐associated disease such as atherosclerosis.^30^, ^36^ Moreover, it has been found that YAP regulates autophagy by actin depolymerization to control proliferation.^17^ Therefore, the level of autophagy was examined in our study, and we found that autophagic flux blockage occurred during YAP promoting senescence, which could be relieved by YAP inhibition in HUVECs and vascular tissues, suggesting that autophagic flux blockage is involved in the promoting effect of YAP on vascular senescence.

Studies over the past decade have uncovered a critical role for mTOR pathways and Hippo pathways as key players regulating organ size through their respective roles in the modulation of cell growth (size) and cell number (proliferation). In addition, the Hippo‐YAP pathway is an upstream regulator of mTOR.^31^, ^37^, ^38^, ^39^ More importantly, increasing mTOR activity perturbs the ability of the whole organism to cope with stress, causing premature senescence and ageing.^37^ Here, we investigated the mTOR signalling alteration in YAP‐promoted senescence, and we found that YAP deficiency decreased mTOR activity while YAP overexpression increased mTOR activity. We next inhibited mTOR activity with rapamycin to ascertain the effects of mTOR on YAP promoting senescence. We found that, by inhibiting mTOR, YAP‐promoted cellular and vascular senescence could be rescued, and the autophagic flux blockage during YAP promoting senescence was also relieved. Collectively, these results illustrated that mTOR signalling pathway plays a vital role in YAP promoting vascular senescence. In addition, YAP‐mTOR may mediate vascular senescence by inhibiting autophagic flux. Furthermore, prior studies have found that mutually regulating effects exist between mTOR and YAP, which could be found in YAP‐promoted senescence here.^16^, ^31^, ^39^, ^40^ Considering the complicated modulation among YAP, mTOR and autophagy, it needs further experiments to prove the clear YAP‐mTOR‐autophagic flux signalling axis.

As far as the existing results are concerned, our study indicated that up‐regulation of YAP could accelerate vascular senescence. In addition, autophagic flux blockage and mTOR activation were observed during YAP‐induced vascular senescence, which could be relieved by YAP inhibition and knockdown. Moreover, YAP‐promoted vascular senescence could be rescued by mTOR inhibition. These findings here provide a novel mechanism of cellular and vascular senescence, and suggest YAP may serve as a potential therapeutic target in ageing‐associated cardiovascular disease treatment.

## CONFLICT OF INTEREST

All authors have no conflict of interest to declare.

## AUTHOR CONTRIBUTION

**Minsheng Chen:** Funding acquisition (equal); Project administration (equal); Supervision (equal). **Caiwen Ou:** Funding acquisition (equal); Project administration (equal); Supervision (equal). **Xianmei Pan:** Conceptualization (equal); Data curation (equal); Writing‐original draft (equal); Writing‐review & editing (equal). **Bo Wu:** Conceptualization (equal); Investigation (equal); Methodology (equal); Resources (equal). **Xianglin Fan:** Formal analysis (equal); Methodology (equal); Writing‐review & editing (equal). **Guanghui Xu:** Formal analysis (equal); Methodology (equal).

## Supporting information

Fig S1

## Data Availability

The data that support the findings of this study are available from the corresponding author upon request.

## References

[1] Widmer RJ , Flammer AJ , Lerman LO , Lerman A . The Mediterranean diet, its components, and cardiovascular disease. Am J Med. 2015;128:229‐238. 25447615 10.1016/j.amjmed.2014.10.014 PMC4339461

[2] Mistriotis P , Andreadis ST . Vascular aging: Molecular mechanisms and potential treatments for vascular rejuvenation. Ageing Res Rev. 2017;37:94‐116. 28579130 10.1016/j.arr.2017.05.006

[3] Papadopoli D , Boulay K , Kazak L , et al. mTOR as a central regulator of lifespan and aging. F1000 Res. 2019;8:F100. 10.12688/f1000research.17196.1 PMC6611156 31316753

[4] Rodier F , Campisi J . Four faces of cellular senescence. J Cell Biol. 2011;192:547‐556. 21321098 10.1083/jcb.201009094 PMC3044123

[5] Shakeri H , Gevaert AB , Schrijvers DM , et al. Neuregulin‐1 attenuates stress‐induced vascular senescence. Cardiovasc Res. 2018;114:1041‐1051. 29528383 10.1093/cvr/cvy059

[6] Fu L , Hu Y , Song M , et al. Up‐regulation of FOXD1 by YAP alleviates senescence and osteoarthritis. PLoS Biol. 2019;17:e3000201. 30933975 10.1371/journal.pbio.3000201 PMC6459557

[7] Iwasa H , Maimaiti S , Kuroyanagi H , et al. Yes‐associated protein homolog, YAP‐1, is involved in the thermotolerance and aging in the nematode Caenorhabditis elegans. Exp Cell Res. 2013;319:931‐945. 23396260 10.1016/j.yexcr.2013.01.020

[8] Guo J , Wu Y , Yang L , et al. Repression of YAP by NCTD disrupts NSCLC progression. Oncotarget. 2017;8:2307‐2319. 27903989 10.18632/oncotarget.13668 PMC5356801

[9] Jin H , Lian N , Bian M , et al. Oroxylin A inhibits ethanol‐induced hepatocyte senescence via YAP pathway. Cell Prolif. 2018;51:e12431. 29318697 10.1111/cpr.12431 PMC6528849

[10] Fausti F , Di Agostino S , Cioce M , et al. ATM kinase enables the functional axis of YAP, PML and p53 to ameliorate loss of Werner protein‐mediated oncogenic senescence. Cell Death Differ. 2013;20:1498‐1509. 23933816 10.1038/cdd.2013.101 PMC3792425

[11] Xie Q , Chen J , Feng H , et al. YAP/TEAD‐mediated transcription controls cellular senescence. Cancer Res. 2013;73:3615‐3624. 23576552 10.1158/0008-5472.CAN-12-3793

[12] Mannaerts I , Leite SB , Verhulst S , et al. The Hippo pathway effector YAP controls mouse hepatic stellate cell activation. J Hepatol. 2015;63:679‐688. 25908270 10.1016/j.jhep.2015.04.011

[13] Huang Y , Pan M , Shu H , He B , Zhang F , Sun L . Vascular endothelial growth factor enhances tendon‐bone healing by activating Yes‐associated protein for angiogenesis induction and rotator cuff reconstruction in rats. J Cell Biochem. 2020;121(3):2343‐2353. 31633245 10.1002/jcb.29457

[14] Wang L , Luo JY , Li B , et al. Integrin‐YAP/TAZ‐JNK cascade mediates atheroprotective effect of unidirectional shear flow. Nature. 2016;540:579‐582. 27926730 10.1038/nature20602

[15] Lv Y , Kim K , Sheng Y , et al. YAP controls endothelial activation and vascular inflammation through TRAF6. Circ Res. 2018;123:43‐56. 29794022 10.1161/CIRCRESAHA.118.313143 PMC6014930

[16] Tumaneng K , Schlegelmilch K , Russell RC , et al. YAP mediates crosstalk between the Hippo and PI(3)K‐TOR pathways by suppressing PTEN via miR‐29. Nat Cell Biol. 2012;14:1322‐1329. 23143395 10.1038/ncb2615 PMC4019071

[17] Pavel M , Renna M , Park SJ , et al. Contact inhibition controls cell survival and proliferation via YAP/TAZ‐autophagy axis. Nat Commun. 2018;9:2961. 30054475 10.1038/s41467-018-05388-x PMC6063886

[18] Frasa MA , Koessmeier KT , Ahmadian MR , Braga VM . Illuminating the functional and structural repertoire of human TBC/RABGAPs. Nat Rev Mol Cell Biol. 2012;13:67‐73. 22251903 10.1038/nrm3267

[19] Kern A , Dikic I , Behl C . The integration of autophagy and cellular trafficking pathways via RAB GAPs. Autophagy. 2015;11:2393‐2397. 26565612 10.1080/15548627.2015.1110668 PMC4835203

[20] Zhang Q , Meng F , Chen S , et al. Hippo signalling governs cytosolic nucleic acid sensing through YAP/TAZ‐mediated TBK1 blockade. Nat Cell Biol. 2017;19:362‐374. 28346439 10.1038/ncb3496 PMC5398908

[21] Narita M , Young AR , Arakawa S , et al. Spatial coupling of mTOR and autophagy augments secretory phenotypes. Science. 2011;332:966‐970. 21512002 10.1126/science.1205407 PMC3426290

[22] Sachdeva K , Do DC , Zhang Y , Hu X , Chen J , Gao P . Environmental exposures and asthma development: autophagy, mitophagy, and cellular senescence. Front Immunol. 2019;10:2787. 31849968 10.3389/fimmu.2019.02787 PMC6896909

[23] Rajendran P , Alzahrani AM , Hanieh HN , et al. Autophagy and senescence: a new insight in selected human diseases. J Cell Physiol. 2019;234:21485‐21492. 31144309 10.1002/jcp.28895

[24] Kuwano K , Araya J , Hara H , et al. Cellular senescence and autophagy in the pathogenesis of chronic obstructive pulmonary disease (COPD) and idiopathic pulmonary fibrosis (IPF). Respir Investig. 2016;54:397‐406. 10.1016/j.resinv.2016.03.010 27886850

[25] Ling Y , Chen G , Deng Y , et al. Polydatin post‐treatment alleviates myocardial ischaemia/reperfusion injury by promoting autophagic flux. Clin Sci. 2016;130(18):1641‐1653. 10.1042/CS20160082 27340138

[26] Bin Z . TEAD mediates YAP‐dependent gene induction and growth control. J Genes Dev. 2008;22(14):1962‐1971. 10.1101/gad.1664408 PMC2492741 18579750

[27] Franceschi C , Campisi J . Chronic inflammation (inflammaging) and its potential contribution to age‐associated diseases. J Gerontol A Biol Sci Med Sci. 2014;69(Suppl 1):S4‐S9. 24833586 10.1093/gerona/glu057

[28] van Steensel B , de Lange T . Control of telomere length by the human telomeric protein TRF1. Nature. 1997;385:740‐743. 9034193 10.1038/385740a0

[29] Totaro A , Zhuang Q , Panciera T , et al. Cell phenotypic plasticity requires autophagic flux driven by YAP/TAZ mechanotransduction. Proc Natl Acad Sci USA. 2019;116:17848‐17857. 31416916 10.1073/pnas.1908228116 PMC6731754

[30] LaRocca TJ , Henson GD , Thorburn A , Sindler AL , Pierce GL , Seals DR . Translational evidence that impaired autophagy contributes to arterial ageing. J Physiol. 2012;590:3305‐3316. 22570377 10.1113/jphysiol.2012.229690 PMC3459044

[31] Liang N , Zhang C , Dill P , et al. Regulation of YAP by mTOR and autophagy reveals a therapeutic target of tuberous sclerosis complex. J Exp Med. 2014;211:2249‐2263. 25288394 10.1084/jem.20140341 PMC4203941

[32] Dong J , Feldmann G , Huang J , et al. Elucidation of a universal size‐control mechanism in Drosophila and mammals. Cell. 2007;130:1120‐1133. 17889654 10.1016/j.cell.2007.07.019 PMC2666353

[33] Muranen T , Selfors LM , Hwang J , et al. ERK and p38 MAPK activities determine sensitivity to pi3k/mtor inhibition via regulation of MYC and YAP. Can Res. 2016;76:7168‐7180. 10.1158/0008-5472.CAN-16-0155 PMC5161652 27913436

[34] De Meyer GR , Grootaert MO , Michiels CF , Kurdi A , Schrijvers DM , Martinet W . Autophagy in vascular disease. Circ Res. 2015;116:468‐479. 25634970 10.1161/CIRCRESAHA.116.303804

[35] Salabei JK , Hill BG . Implications of autophagy for vascular smooth muscle cell function and plasticity. Free Radic Biol Med. 2013;65:693‐703. 23938401 10.1016/j.freeradbiomed.2013.08.003 PMC3859773

[36] LaRocca TJ , Gioscia‐Ryan RA , Hearon CM Jr , Seals DR . The autophagy enhancer spermidine reverses arterial aging. Mech Ageing Dev. 2013;134:314‐320. 23612189 10.1016/j.mad.2013.04.004 PMC3700669

[37] Iglesias‐Bartolome R , Gutkind JS . Signaling circuitries controlling stem cell fate: to be or not to be. Curr Opin Cell Biol. 2011;23:716‐723. 21880478 10.1016/j.ceb.2011.08.002 PMC3391582

[38] Csibi A , Blenis J . Hippo–YAP and mTOR pathways collaborate to regulate organ size. Nat Cell Biol. 2012;14:1244‐1245. 23196842 10.1038/ncb2634

[39] Hu JK , Du W , Shelton SJ , Oldham MC , DiPersio CM , Klein OD . An FAK‐YAP‐mTOR signaling axis regulates stem cell‐based tissue renewal in mice. Cell Stem Cell. 2017;21(1):91‐106.e6. 28457749 10.1016/j.stem.2017.03.023 PMC5501749

[40] Hansen CG , Ng YL , Lam WL , Plouffe SW , Guan KL . The Hippo pathway effectors YAP and TAZ promote cell growth by modulating amino acid signaling to mTORC1. Cell Res. 2015;25:1299‐1313. 26611634 10.1038/cr.2015.140 PMC4670996

