# Supplementary material for: YAP accelerates vascular senescence via blocking autophagic flux and activating mTOR
Source: J Cell Mol Med. 2020 Dec 12;25(1):170–83. doi: 10.1111/jcmm.15902 (PMC7810949; doi:10.1111/jcmm.15902)
Supplement: Supplementary file 1 — Fig S1 [file JCMM-25-170-s001.docx]

**
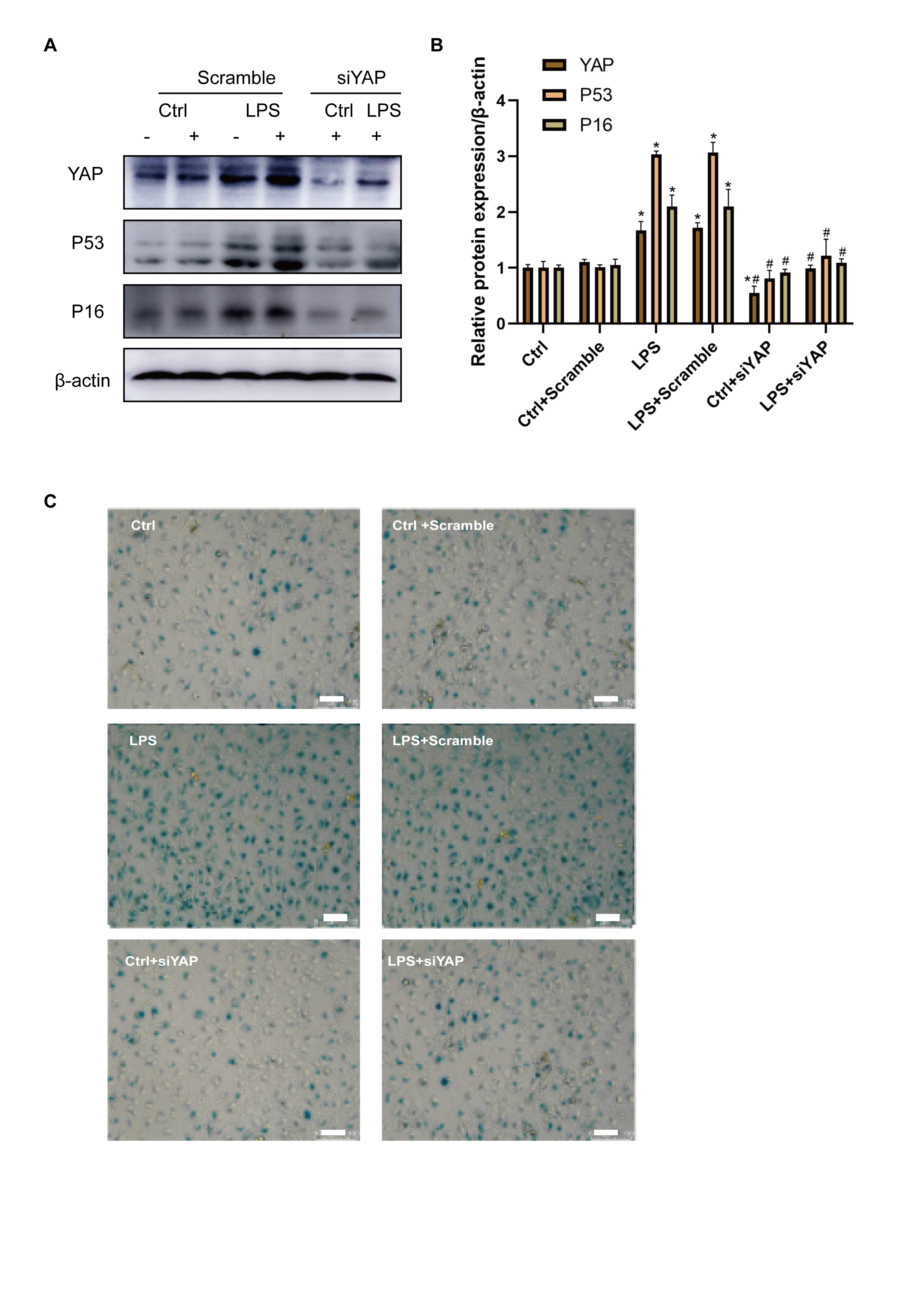
Figure 1** (A) Western Blot was preformed to analyze YAP, P16 and P53 in cells transfected with YAP siRNA (siYAP#1, siYAP#2, siYAP#3) or negative control siRNA for 6 hours. (B) The semi-quantification of the proteins in panel A respectively. (C) SA-β-gal staining was used to analyze senescence degree in HUVECs transfected with YAP siRNA (siYAP#1, siYAP#2, siYAP#3) or negative control siRNA(non-targeting 20-25nt siRNA) for 6 hours; scale bar=100μm. All experiments were repeated at least three times and data are expressed as mean ± SEM, **p*<0.05 vs. Ctrl group, #*p*<0.05 vs. LPS group.
